# Regulatory Role of N6-Methyladenosine in Longissimus Dorsi Development in Yak

**DOI:** 10.3389/fvets.2022.757115

**Published:** 2022-04-13

**Authors:** Xiaoming Ma, Yongfu La, Pengjia Bao, Min Chu, Xian Guo, Xiaoyun Wu, Jie Pei, Xuezhi Ding, Chunnian Liang, Ping Yan

**Affiliations:** ^1^Lanzhou Institute of Husbandry and Pharmaceutical Sciences, Chinese Academy of Agricultural Sciences, Lanzhou, China; ^2^Gansu Provincial Key Laboratory of Yak Breeding Engineering, Lanzhou Institute of Animal Husbandry and Veterinary Medicine, Chinese Academy of Agricultural Sciences, Lanzhou, China

**Keywords:** yak, longissimus dorsi, MeRIP-Seq, transcriptional regulation, muscle development

## Abstract

N6-methyladenine (m6A) RNA undergoes epigenetic modification, which is the most extensive intermediate chemical modification in mRNA. Although this modification occurs in all living organisms, it is the most widely studied among mammals. However, to date, no study has investigated the m6A transcriptome-wide map of yak and its potential biological functions in muscle development. In this study, the differences of m6A methylation and gene expression in yak muscle development belonging to three age groups, namely 3 years (group A), 6 months (group M), and 90-day-old fetuses (group E), were determined by using methylated RNA immunoprecipitation sequencing (MeRIP-Seq) and RNA sequencing (RNA-Seq). In these three groups, a total of 6,278 (A), 9,298 (E), and 9,584 (M) m6A peaks were identified, with average densities between 1.02 and 2.01. m6A peaks were mostly enriched in the stop codon, 3′ untranslated region (UTR) region, and inner long exon region with consensus motifs of UGACA. In all the three stages, the m6A peak enrichment level was significantly negatively correlated with mRNA abundance (Pearson's correlation coefficient *r* = −0.22 to −0.32, *p* < 10^−16^). The functional enrichment of genes consistently modified by m6A methylation, particularly those genes that regulate cell differentiation as well as muscle growth and development, was observed at all three stages. Moreover, m6A abundance was negatively associated with gene expression levels, indicating that m6A might play a vital role in modulating gene expression during yak muscle development. This comprehensive map thus provides a solid foundation for determining the potential functional role of m6A RNA modification in yak muscle growth.

## Introduction

Yak (*Bos grunniens*), referred to as “the boat of the plateau,” mainly inhabits the Qinghai–Tibet plateau and its adjacent alpine or subalpine areas with an altitude of 3,000–5,000 m (1). Yaks exhibit strong adaptability to high altitude, cold resistance, and rough feeding tolerance, which makes them a crucial source of livelihood for farmers and herdsmen on the Qinghai–Tibet plateau. An improvement has been made in some domesticated varieties (strains) primarily for their milk, meat, and as beasts of burden (2). Yak meat is delicious, low in fat, and high in protein; it is rich in many vital vitamins and minerals (3). However, the growth of yak is slow, and the quality of its meat is limited by muscle retardation, low-fat deposition efficiency, poor tenderness, and rough taste, which are not conducive to the production and development of yak products in the Qinghai–Tibet plateau.

RNA methylation is a complex and diverse chemical modification in mRNA. It accounts for >60% of more than 150 chemical RNA modifications and various methylation modifications and has diverse biological functions in cells (4). N6-methyladenine (m6A) in RNA (N6-methyladenosine) is formed when methyltransferase catalyzes the transfer of methyl on the sixth N atom of adenine by acting on the donor S-adenosine methionine (S-adenosyl methionine). Methyltransferases (such as METTL3, METTL14, WTAP, KIAA1429, and -CH3) perform m6A methylation of adenine, which is removed under the catalytic action of demethylases (such as FTO and ALKBH5) (5). m6A is the most predominant RNA methylation modification in eukaryotic mRNA (6) and has been extensively studied in recent years. m6A modification involves almost all the aspects of RNA metabolism. The continuous discovery of recognition proteins (readers) indicates that m6A modification participates in various RNA regulations. m6A regulates the fate of RNA through readers, which are involved in precursor RNA splicing, RNA transport outside the nucleus, RNA stability, and mRNA translation (7–10). Various regulatory functions of m6A in RNA metabolism indicate its role in the regulation of various biological processes, including tissue development.

Recent studies on energy metabolism and fat deposition have investigated the effects of m6A modification on animal growth and development. The overexpression and specific knockout of *FTO* and *METTL3* genes in porcine adipose cells indicated that the FTO expression level was negatively correlated with the m6A level and positively correlated with adipogenesis, whereas the METTL3 expression level was positively correlated with the m6A level and negatively correlated with adipogenesis (11). Mice deficient in FTO function exhibited increased energy consumption, lean body size, and severe growth retardation and deformities (12). This mice study also found that Irx3 is a functional long-range target of obesity-associated variants within the FTO gene, and both Irx3 and FTO interact, increasing body fat deposition and leading to obesity among animals. Irx3 deficiency in FTO mice reduces fat deposition by increasing the energy metabolism rate (13). These results reveal that m6A is vital for energy metabolism and fat deposition in animals. Currently, research on the role of m6A RNA modifications in muscle development is still in the initial stage. Myoblasts are a crucial research model to study skeletal muscle proliferation and differentiation, in which myoblasts form multinucleated myotubules under induced conditions (14). Kudou and Chen confirmed that METTL3 regulates myoblast differentiation and m6A modifies *MyoD* expression (15, 16). m6A-modified genes in the skeletal muscle of wild boar and in Changbai and Rongchang pigs are interspecific, and these genes tare mainly enriched in transcription factor binding and cell metabolism (17). These results indicate that m6A plays a pivotal regulatory role in the skeletal muscle; however, its regulatory role and mechanism in skeletal muscle development remain unclear.

In this study, m6A functions were further investigated to facilitate future studies on mammalian m6A. Three skeletal muscle tissues of developing yak were collected, and the distribution pattern of m6A in the developmental transcriptome as well as the relationship between m6A modification and gene expression were analyzed. Various m6A-modified genes have been found to have biological functions in the three developmental stages. These results provide resources for identifying the mRNA modified by adenosine methylation in muscles and expand our understanding of the role of m6A in the development and growth of mammalian organs.

## Materials and Methods

### Animals and Tissue Collection

Yaks were selected from the livestock in the Ashidan Mountain area of the Qinghai province. All the yaks were raised at the same farm by using the same feeding and management practices. A total of nine healthy female yaks belonging to three age groups, namely 6 months (group M), 3 years (group A), and 90-day fetuses (group E), were selected. Fetal age was estimated from the crown–rump length (18). Study samples [longissimus dorsi (LD)] were obtained after slaughtering animals. For RNA isolation, the samples were divided into 0.5 cm^3^ portions, treated with RNA later (Qiagen, Hilden, Germany), and stored overnight at 4°C before being stored in liquid nitrogen. All the yaks were handled in strictly accordance with the Animal Ethics Procedures and Guidelines of the People's Republic of China. This study was approved by the Animal Administration and Ethics Committee of Lanzhou Institute of Husbandry and Pharmaceutical Sciences of the Chinese Academy of Agricultural Sciences (Permit No. 2019-002).

### RNA Isolation and Fragmentation

Total RNA was isolated from the LD tissue using the TRIzol reagent (Invitrogen Co., CA, USA) according to the manufacturer's instructions. The rRNA content of the total RNA was reduced using the Ribo-Zero rRNA Removal kit (Illumina Inc., CA, USA), and RNA was chemically fragmented into ~100-nucleotide-long fragments using the fragment buffer (Illumina, Inc.).

### Library Preparation and High-Throughput Sequencing

Methylated RNA immunoprecipitation sequencing (MeRIP-Seq) was performed by OE Biotech Co. (Shanghai, China). Briefly, m6A RNA immunoprecipitation (IP) was performed according to the instructions of the GenSeqTM m6A RNA IP kit (GenSeq, Inc., China), and the immunoprecipitated RNA and input RNA libraries were constructed using the NEBNext 1 Ultra™ RNA Library Prep kit of Illumina 1 (E7530L, New England Biolabs). Library quality was evaluated using the BioAnalyzer 2100 system (Agilent Technologies Inc., USA), and the library was sequenced on an Illumina HiSeq instrument (Illumina Corp.) with 150-bp paired-end reads.

### Data Analysis

Adaptor and low-quality bases were trimmed using Trimmomatic (version: 0.39) (19), and SortMeRNA (20) software was used for removing rRNA reads. After discarding rRNA reads, the remaining clean reads were mapped to the reference genome (LU_Bosgru_v3.0) by using HISAT2 (21) with default parameters, and unique reads with high mapping quality were retained.

The Guitar R package (22) and deepTools (23) software were used for evaluating the quality of the MeRIP-Seq data. m6A-enriched peaks in each m6A IP sample were identified using MeTDiff (24) peak calling software where the corresponding input sample served as a control. MeTDiff was run with the options (“FRAGMENT_LENGTH = 200, PEAK_CUTOFF_PVALUE = 0.01, PEAK_CUTOFF_FDR = 0.05”) for peak detection. The called peaks were annotated by intersecting with the gene architecture using ChIPseeker software (25). The sequence motif was detected using MEME (26) and DREME (27) software. Then, the motifs were annotated using Tomtom software.

Differential analysis of the MeRIP-Seq data led to the identification of the differences in the RNA methylome in a case-control study (A vs. E, M vs. A, and M vs. E), differential peaks were detected using MeTDiff with the parameter (“FRAGMENT_LENGTH = 200, PEAK_CUTOFF_PVALUE = 0.01, DIFF_PEAK_CUTOFF_FDR = 0.05, PEAK_CUTOFF_FDR = 0.05”). Then, differential peaks were annotated using ChIPseeker. The gene ontology (GO) enrichment and Kyoto encyclopedia of genes and genomes (KEGG) pathway enrichment analyses of peaks and differential peaks were performed using DAVID (https://david.ncifcrf.gov/), based on the hypergeometric distribution.

In RNA sequencing (RNA-Seq), differences in gene expression were categorized as upregulation and downregulation. In MeRIP-Seq, the downregulation of gene methylation was noted according to the changes in peak abundance. In this study, correlation analyses for the two omics contents were performed using the Python (v.2.7.12) script to simultaneously compare transcription and methylation levels.

### Quantitative Real-Time PCR

To study the m6A status in the yak LD tissue at different stages, the levels of RNA methylation-related genes, such as *YTHDF2, RBM15B, RBM15, RB1, METTL16, FTO, HES1, FOS, DNAJB2, COPS2*, and *ALKBH5*, were detected through quantitative real-time polymerase chain reaction (qRT-PCR). Details of the primers designed to amplify these 11 genes have been presented in Supplementary Table 1; the β*-actin* gene was used as an internal reference. Real-time PCR was performed using the LightCycler® 480 II Real-time PCR Instrument (Roche, Swiss) with 10-μl PCR reaction mixture that included 1 μl of cDNA, 5 μl of 2× *PerfectStart*^TM^ Green qPCR SuperMix, 0.2 μl of forward primer, 0.2 μl of reverse primer, and 3.6 μl of nuclease-free water. The reaction mixture was incubated in a 384-well optical plate (Roche, Swiss) at 94°C for 30 s, followed by 45 cycles of 94°C for 5 s and 60°C for 30 s. mRNA expression levels were normalized to input the reference gene and were calculated using the 2^−Δ*ΔCt*^ method.

## Results

### Transcriptome-Wide Mapping of the m6A Methylation Landscape in Yak LD Tissues

The MeRIP-Seq produced 27.65–48.07 M raw reads from the input or immunoprecipitated LD tissues of groups M, A, and E yaks. After filtering out the low-quality data, more than 54,545,026 high-quality reads from each sample were mapped to the LU_Bosgru_v3.0 genome, and then more than 79.57% of the clean reads from all samples were uniquely mapped to the yak reference genome (Supplementary Table 2). The Q30 base distribution ranged from 93.85 to 94.97%, and the average GC content was 54.65%. The number of peaks detected in each group was as follows: A: 6,278; E: 9,298; and M: 9,584 (Supplementary Tables 3, 4). This study concomitantly showed that among all m6A-modified genes in the LD transcriptome, the density of m6A peaks averaged between 1.02 and 2.01 (Supplementary Table 4).

**Figure 1:**
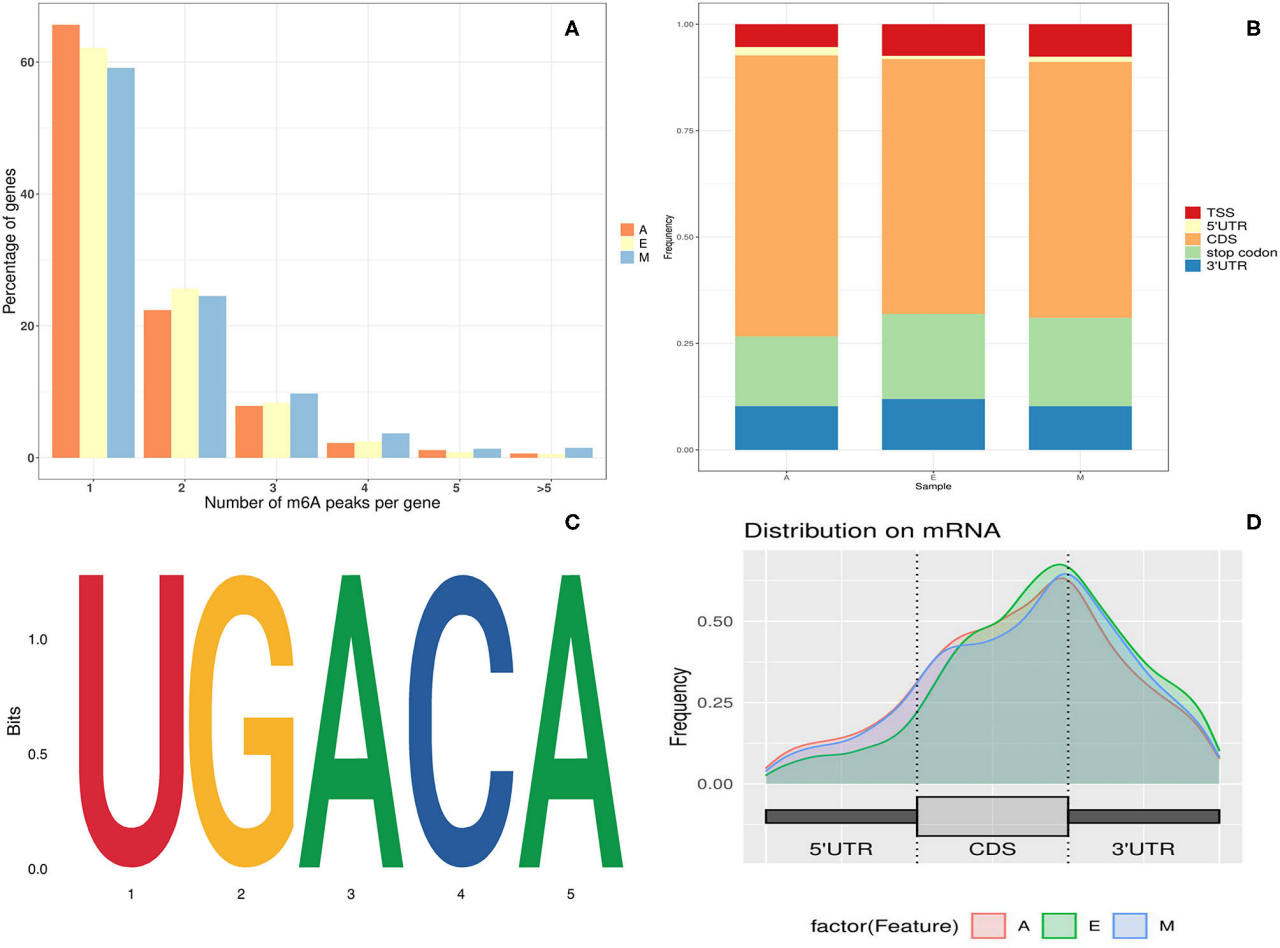
Overview of N6-methyladenine (m6A) methylation in yak longissimus dorsi (LD). **(A)** Proportion of genes containing variant numbers of m6A peaks. **(B)** A graphical representation of the frequency of m6A peaks in five non-overlapping segments in the three developmental stages. **(C)** Motif sequence containing m6A. **(D)** Distribution of summits of m6A peaks along with transcripts.

The analysis of the number of m6A peaks revealed that most (86.49%) m6A-modified single gene transcripts of LD transcription contained at least one or two m6A peaks. The number of genes with more than three modification sites accounted for ~13.51% of the total number of modified genes (Figure 1A). This was lower than the proportion of genes with multiple modification sites, which was previously reported in the human brain (16.70%) (28).

Regarding m6A peak distribution, m6A peaks in animals tend to be enriched near the stop codon, 3′ untranslated region (UTR) region, and inner long exon region (Figure 1B). On the contrary, in plants, m6A peaks are enriched not only near the start codon region but also in the start codon region. To understand the preferred methylation sites of m6A modification in yak transcriptome, the m6A peak was then investigated in the total transcriptome of yak LD tissues. The m6A peak region detected here was found to be associated with the Lu_bosgru_v3.0 genome reference annotation region in the Yak Ensemble database. Peak localization changed from genome-wide peak localization to transcriptome-wide peak localization.

The m6A peak was further allocated to five non-overlapping gene transcripts [i.e., 5′ UTR region, continuous deflective separation (CDS) region, stop codon region, 3′ UTR region, and the transcription start site (TSS) region] to systematically evaluate the accurate distribution of m6A modification sites in mRNA. As evident in Figure 1D, ~80% of m6A peaks were distributed within the known gene interval, more than 60% of the peaks were distributed near CDS and stop codon regions, and ~30% of m6A peaks were distributed in the 5′ UTR, start codon, and 3′ UTR regions. Moreover, the distribution trends of the three age groups were highly similar, suggesting that the classical recognition sequence of m6A methylation was conserved in animal tissues.

To determine whether the m6A peak identified in this experiment contained the classical m6A conserved sequence, RRACH (*R* = G, *A* = m6A, *H* = non-G), a routine search on the motif of the m6A peak enrichment region was performed. The Homer (NC, 45) analysis software was used to find the conserved motif sequence in the m6A peak enrichment Top1000 sequence and around each 50-bp region of the peak. The cluster sequence of the complete RRACH classical conserved sequence of m6A was therefore found to be significantly enriched in the yak LD at different growth stages (Figure 1C) Common UGACA sequences were identified in groups E, M, and A (*p*-values of the three groups were A: 1e-161; E: 1e-255; and M: 1e-272, respectively). The identification of these conserved sequences laid a solid foundation for the authenticity of m6A modification, and further confirmed the existence of the modification function of m6A methylation in the yak LD.

### Relationship Between m6A Modification and Gene Characteristics and Expression

**Figure 2:**
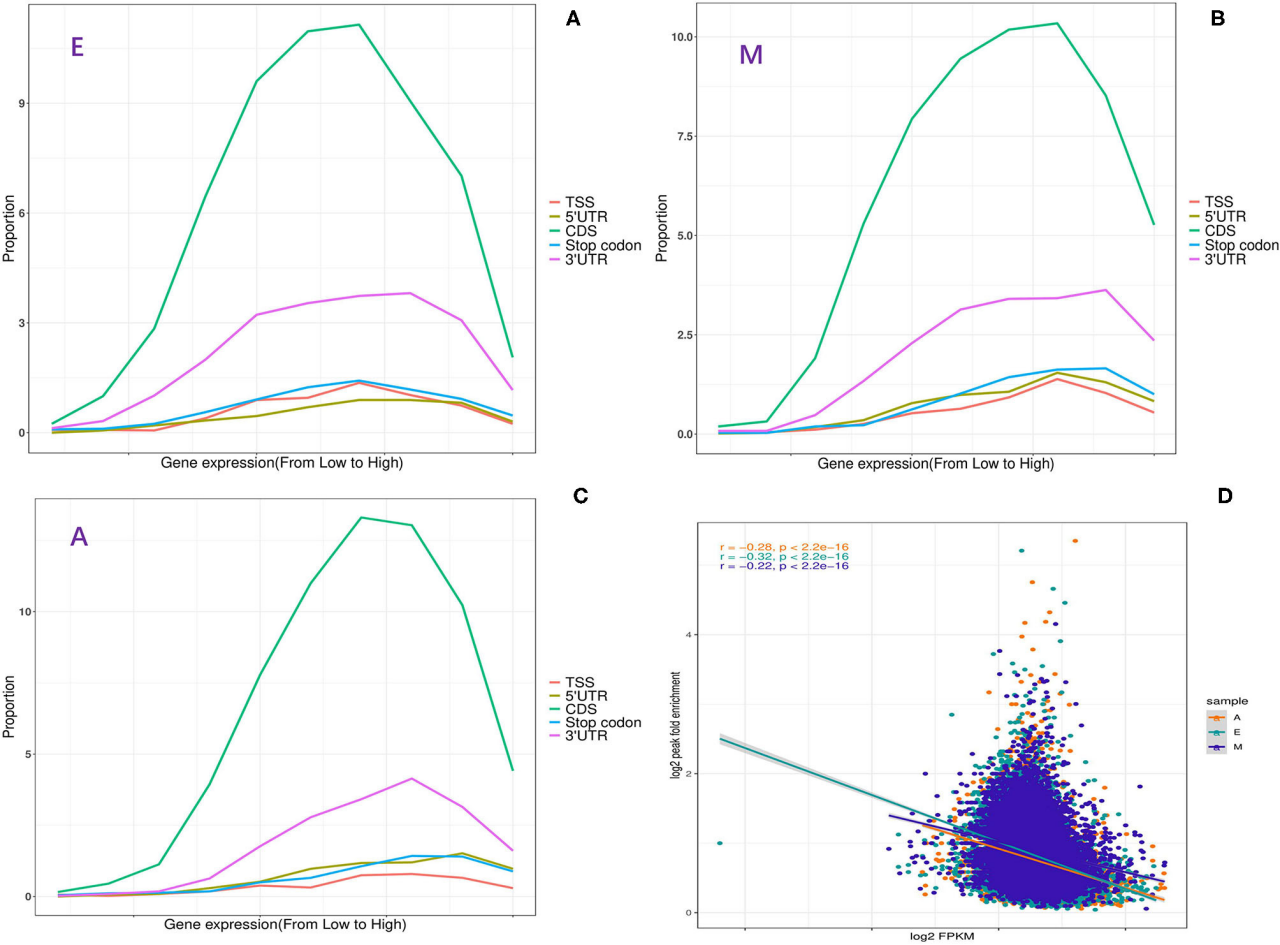
Relationship between m6A methylation and expression of modified genes. **(A–C)** Fraction of genes with m6A peaks in each of the segments as a function of the expression level. **(D)** The plot of m6A peak enrichment and mRNA abundance in the three developmental stages.

Furthermore, to determine whether m6A RNA modification in LD has a potential regulatory relationship with the expression level of a m6A-modified gene, the functional relationship curve between the m6A peak in each segment and expression level was drawn (Figures 2A–C). Each segment displayed a non-monotonic pattern of functional relationship, which was consistent with the relationship between m6A modification and gene expression in mice and humans (29). This non-monotonic functional relationship indicated that most m6A-modified genes have moderate expression levels (Figures 2A–C). Moreover, in this study, m6A peak enrichment levels in three stages were significantly negatively correlated with mRNA abundance (Pearson's correlation coefficient *r* = −0.22 to −0.32, *p* < 10^−16^) (Figure 2D). This was consistent with the results of previous reports (28, 30). Therefore, it was speculated that differences in the m6A modification level were related to the genetic structural characteristics of the modified genes.

### m6A-Modified Genes Participate in the Regulation of Important Biological Functions

**Figure 3:**
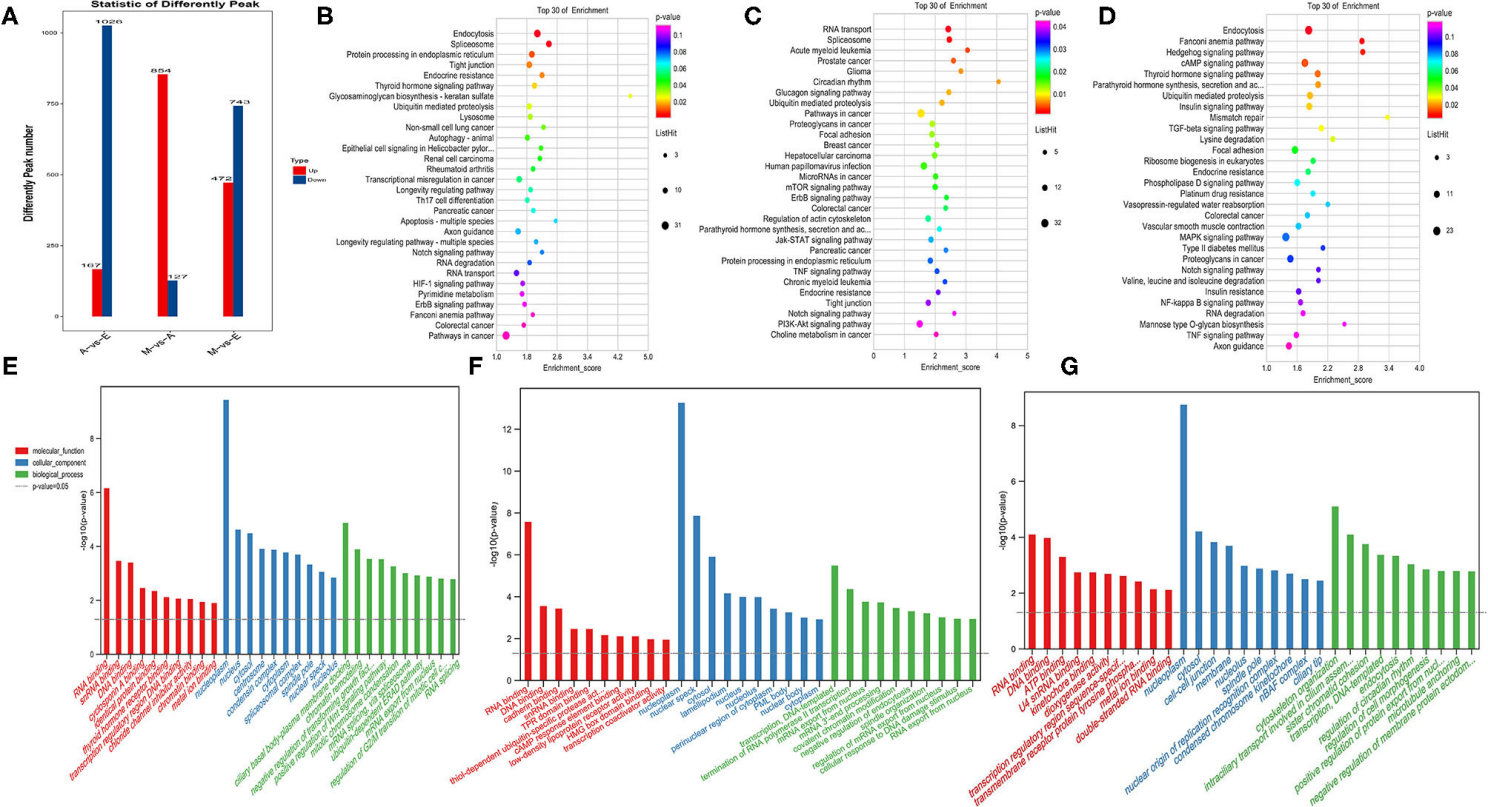
Enrichment analysis of m6A-modified genes in each group. **(A)** The number of genes showed differential m6A methylation. **(B–D)** Kyoto encyclopedia of genes and genomes (KEGG) analysis in AvE, MvA, and MvE. **(E–G)** Gene ontology (GO) analysis in AvE, MvA, and MvE.

The peak abundance of N6-methyladenine in each group was compared. In the A vs. E group, 167 markedly hypermethylated m6A peaks and 1,026 substantially hypomethylated peaks were observed (Figure 3A). In addition, the m6A peak of differential methylation represented the genes studied by GO and KEGG pathway analyses, and highlighted the biological significance of m6A methylation in yak LD development. The GO analysis revealed differentially methylated genes mainly implicated in the nucleoplasm (ontology: cellular_component), RNA binding (ontology: molecular_function), and ciliary basal body–plasma membrane docking (ontology: biological_process) (Figure 3E and Supplementary Table 5). Meanwhile, the top 30 bioenriched candidates of the KEGG pathway were differentially methylated genes significantly correlated with endocytosis, a tight junction, and the notch signaling pathway (Figure 3B and Supplementary Table 5). In the M vs. A group, the findings revealed 854 markedly hypermethylated m6A peaks and 127 substantially hypomethylated peaks (Figure 3A). The GO analysis revealed differentially methylated genes mainly implicated in the nucleoplasm (ontology: cellular_component), RNA binding (ontology: molecular_function), and transcription, DNA-templated (ontology: biological_process) (Figure 3C and Supplementary Table 5). Meanwhile, the top 30 bioenriched candidates of the KEGG pathway were differentially methylated genes significantly correlated with RNA transport, focal adhesion, the mammalian target of rapamycin (mTOR) signaling pathway, and the PI3K-Akt signaling pathway (Figure 3F and Supplementary Table 5). In the M vs. E group, the findings revealed 472 markedly hypermethylated m6A peaks and 743 substantially hypomethylated peaks (Figure 3A). The GO analysis revealed differentially methylated genes mainly implicated in the nucleoplasm (ontology: cellular_component), RNA binding (ontology: molecular_function), and cytoskeleton organization (ontology: biological_process) (Figure 3D and Supplementary Table 5). Meanwhile, the top 30 bioenriched candidates of the KEGG pathway were differentially methylated genes significantly correlated with the insulin signaling pathway, the Hedgehog signaling pathway, focal adhesion, and the transforming growth factor-beta (TGF-beta) signaling pathway (Figure 3G and Supplementary Table 5).

**Figure 4:**
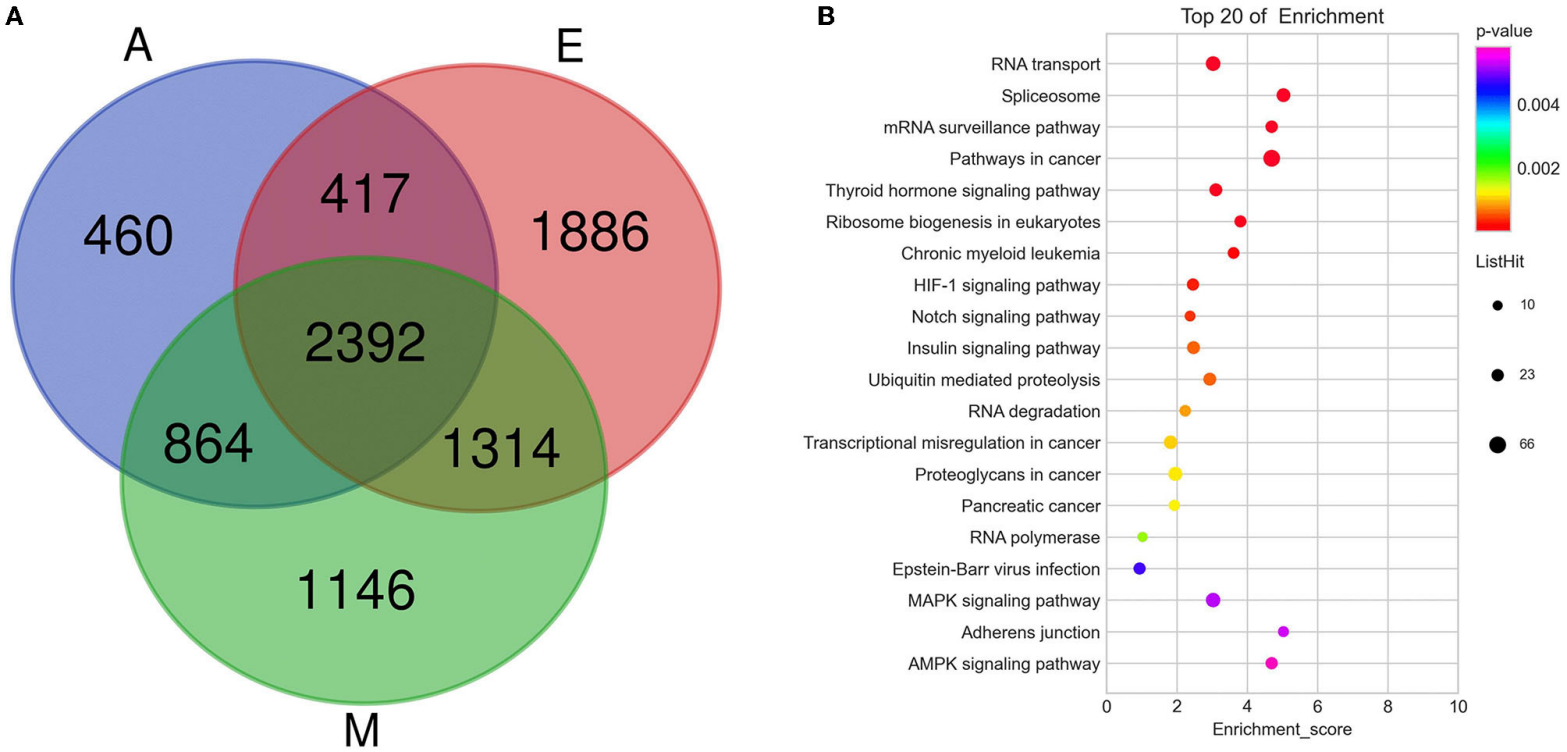
The consistently modified genes in yak LD enrich the analysis. **(A)** Venn diagram showed the overlap of m6A peaks in the three developmental stages; **(B)** KEGG enrichment of consistently modified genes in yak LD in the three developmental stages.

To study the important regulatory function of m6A modification in the yak LD, DAVID (https://david.ncifcrf.gov/) was used for analyzing the gene function enrichment of 2,392 genes (Figure 4A) that were continuously modified by m6A in three age groups. Function-based enrichment analysis revealed these persistent m6A-modified genes to be significantly (*p* < 0.05, Benjamini Hochberg) enriched in important biological function regulation pathways: RNA polymerase (*p* = 0.0017), RNA transport (*p* = 3.46E-9), and RNA degradation (*p* = 8.56E-4) (Figure 4B and Supplementary Table 6). Furthermore, some m6A-modified genes were involved in regulating cell differentiation and muscle growth and development, including the Notch (*p* = 4.05E-4), insulin (*p* = 5.69E-4), and mitogen-activated protein kinase (MAPK) signaling pathways (*p* = 0.0052) (Figure 4B and Supplementary Table 6).

### A Conjoint Analysis of MeRIP-Seq and RNA-Seq Data With Both the Groups

**Figure 5:**
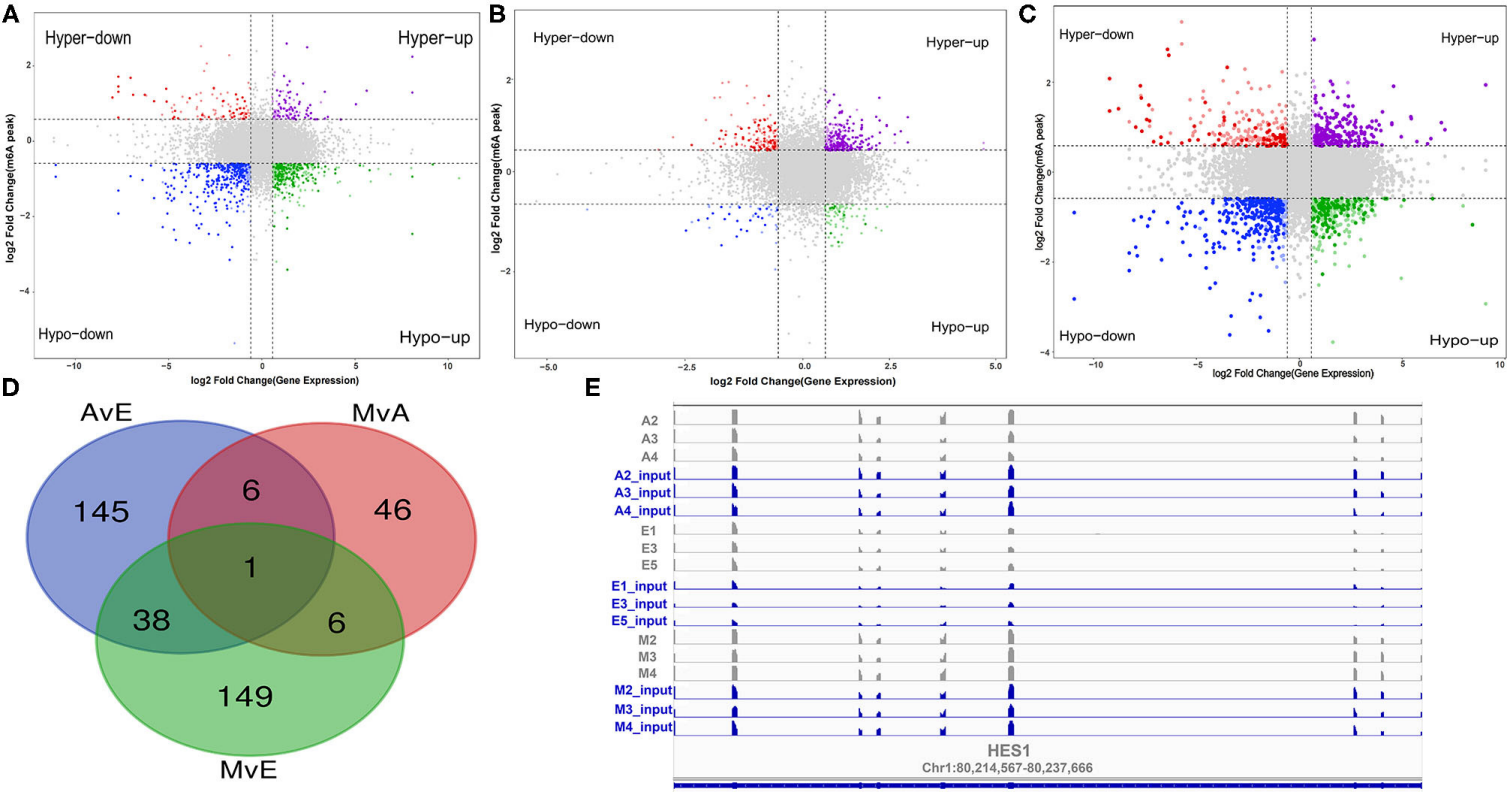
A conjoint analysis of methylated RNA immunoprecipitation sequencing (MeRIP-Seq) and RNA sequencing (RNA-Seq) data. **(A–C)** Distribution of genes with a significant change in both the m6A methylation level and gene expression in each group. **(D)** Venn diagram showed the negative correlation of m6A-modified genes in each group. **(E)** m6A enrichment and gene expression profile of HES1 in the three stages.

A cross-analysis of MeRIP-Seq and RNA-Seq results revealed an interesting relationship between differentially methylated m6A peaks and gene expression patterns in muscle development. A negative correlation was observed between the differentially methylated m6A peaks and gene expression levels (Figures 5A–C). Otherwise, all the genes were segregated into four main types: hypermethylated and upregulated genes termed as “hyper-up,” hypomethylated and downregulated genes termed as “hypo-down,” hypermethylated and downregulated genes termed as “hyper-down,” and hypomethylated and upregulated genes termed as “hypo-up.” According to the relationship between m6A methylation and gene modification, two modes were selected: hyper-down and hypo-up. The A vs. E group had 154 hypo-up and 36 hyper-down genes, the M vs. A group had 9 hypo-up and 50 hyper-down genes, and the M vs. E group had 118 hypo-up and 76 hyper-down genes. The Venn diagram of the negative correlation between the aforementioned three developmental stages (Figure 5D) showed that the three comparison groups had only one gene at the intersection (HES1). The level of m6A modification in each group was higher than that of transcript expression, and a negative regulatory modification was noted (Figure 5D).

**Figure 6:**
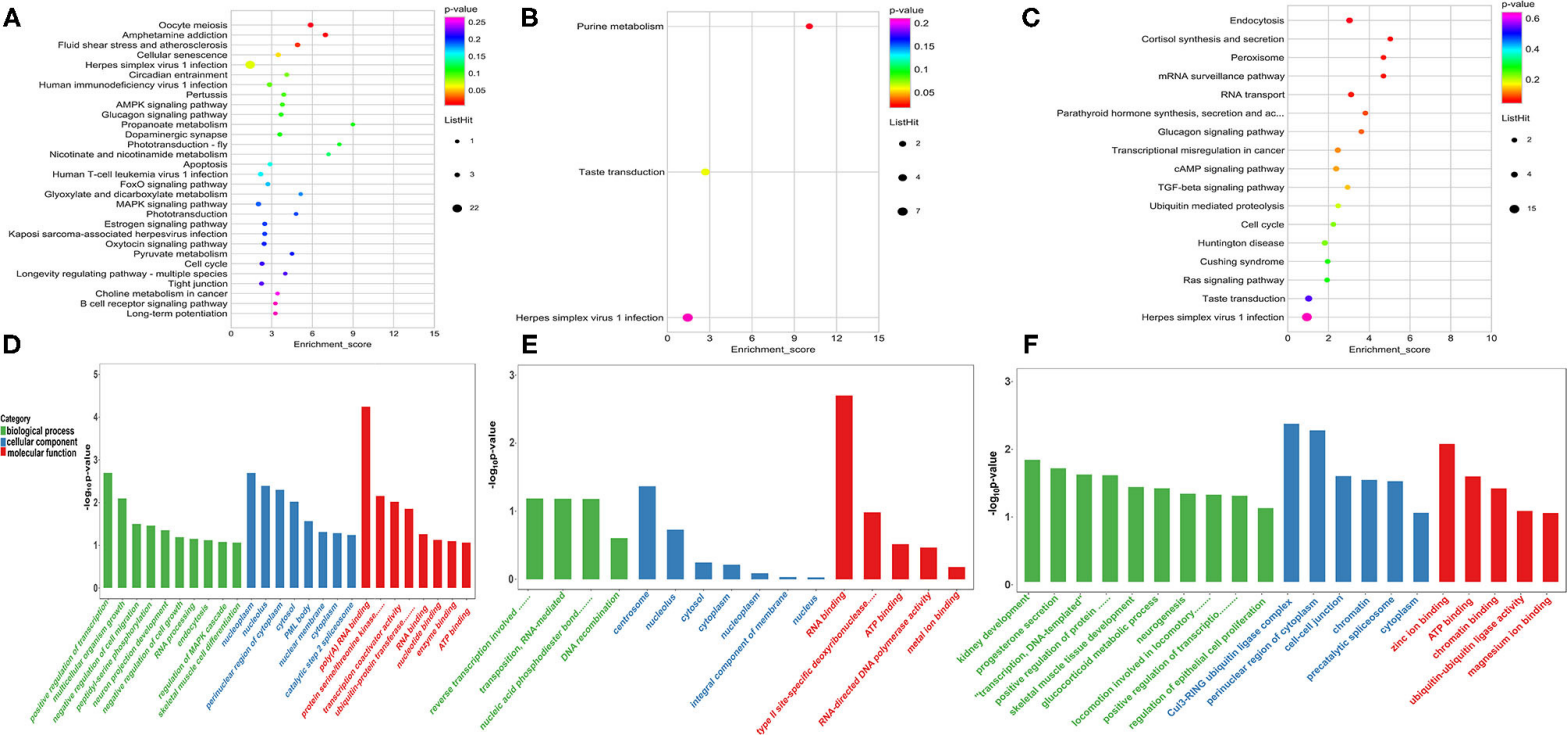
Enrichment analysis of the negatively correlated modified genes in each group. **(A–C)** The KEGG analysis in AvE, MvA, and MvE. **(D–F)** The GO analysis in AvE, MvA, and MvE.

Furthermore, negatively correlated modified genes in each group were analyzed for their functional enrichment. In the A vs. E group, 190 negatively correlated modified genes were enriched in 30 KEGG pathways, of which only three were significantly enriched (*p* < 0.05). The results indicated that these genes were enriched in multiple processes, such as the AMP-activated protein kinase (AMPK) signaling pathway, apoptosis, and the FOXO signaling pathway. The GO analysis of these genes revealed that they were involved in poly(A) RNA binding, nucleoplasm, the growth of multicellular organisms, and skeletal muscle cell differentiation (Figures 6A,D and Supplementary Table 7). In the M vs. A group, 58 negatively correlated modified genes were enriched in three KEGG pathways, of which only one was significantly enriched (*p* < 0.05) (purine metabolism). The GO analysis of these genes revealed their involvement in RNA binding, RNA splicing, and endoplasmic reticulum lumen (Figures 6B,E and Supplementary Table 7). In the M vs. A group, 194 negatively correlated modified genes were enriched in 17 KEGG pathways, of which only one was significantly enriched (*p* < 0.05) (endocytosis). The GO analysis of these genes revealed their involvement in skeletal muscle tissue development, perinuclear region of the cytoplasm, and protein serine/threonine kinase activity (Figures 6C,F and Supplementary Table 7).

### Verification of m6A-Modified Differentially Expressed Genes

**Figure 7:**
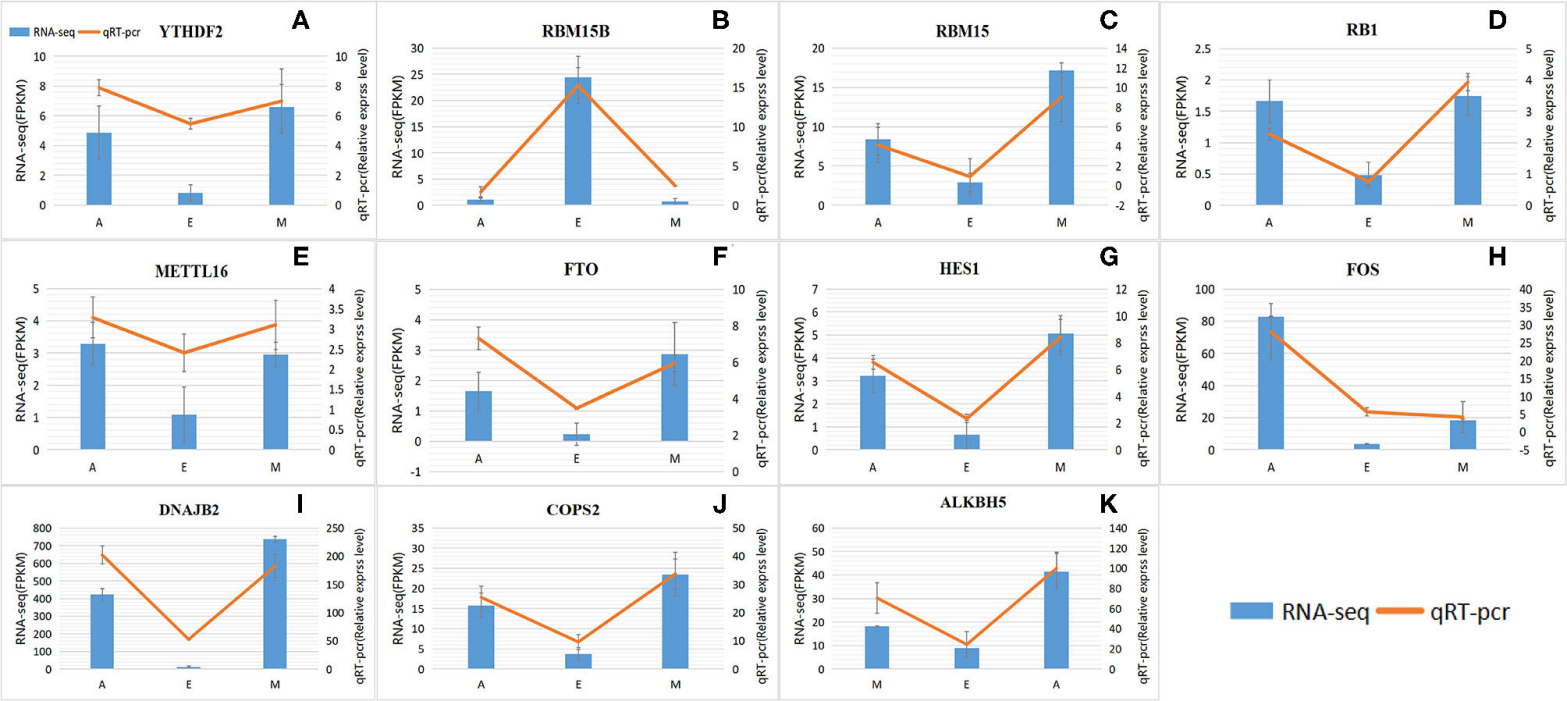
Levels of RNA methylation-related genes validated by quantitative real-time polymerase chain reaction (qRT-PCR). **(A–K)** RNA-Seq results are shown as a bar graph. The value to the right of the *y*-axis represents FPKM. The qRT-PCR results are shown in the line graph, with the *y*-axis on the left representing the relative expression level. Data represent mean ± standard error (SE).

These results were consistent with RNA-Seq data, which confirmed the reliability of the sequencing data (*R* = 0.92, *p* < 2.2e-16) (Figure 7 and Supplementary Figure 1). These results further revealed the identity and relationships among the target genes, confirming the validity of our transcriptome analysis.

## Discussion

N6-methyladenine is a highly conserved but widespread epigenomic modification in mammalian mRNA (31). Due to the lack of knowledge regarding m6A methylation in yaks, a study investigated the modification pattern of m6A methylation in yaks. We reported comprehensive transcriptome-wide patterns of m6A in the yak LD tissues based on the previously reported MeRIP-Seq method (29). Consequently, the m6A modification profile of the entire transcriptome was identified in pig muscle and adipose tissues for the first time. Moreover, methylation modification sites in yaks were identified to be enriched mainly around the stop codon, CDS, TSS, and 3′ UTR regions, and to some extent, in the 5′ UTR region. These reports were similar to previous reports in humans, pigs, and mice, which stated that m6A modification was mainly enriched in the inner exon, near the stop codon, 3′ UTR region, and 5′ UTR region (28). These results thus indicated that the overall distribution of m6A modification sites in the mammalian transcriptome is similar, reinstating that m6A modifications are conserved among species. Highly enriched m6A at the stop codon or 3′ UTR region might be due to their role in RNA stability, signal transduction, and signal transfer, or as regulatory elements of protein translation through the recruitment of specific factors at the m6A site for RNA transport or protein synthesis (7). Studies have suggested that the consistent motif pattern of “RRACH” is over-represented in the m6A motif sequence area (29, 32). Accordingly, compared with previous studies (28, 29), the consensus motif of the UGACA sequence in the yak LD tissue transcriptome was appropriately identified in this study, revealing that RNA adenosine methylation was conservative in mammals.

Most m6A-modified genes are negatively correlated with the enrichment of the m6A peak (Pearson's correlation coefficient *r* = −0.22 to −0.32, *p* < 10^−16^). m6A is a chemical marker associated with transcriptome conversion. Compared with non-m6A-labeled transcripts, m6A-labeled transcripts possess a significantly shorter RNA half-life and higher mRNA decay rate (33). At the same time, high m6A methylation levels might confer high RNA stability on low-expressing transcripts or signal binding capacity of the reader proteins (34). In our study, 34% of m6A-modified genes exhibited more than two m6A-modified sites, which also likely increases RNA stability or the probability of recognizing the reader protein. All these results indicated that m6A is modified to mediate transcriptional regulation in the LD transcript of yaks.

The genes consistently modified by m6A at three ages were related to muscle cell differentiation and growth. Studies have revealed similar results. For example, a m6A-modified gene in the embryonic stem cell transcriptome is involved in the regulation of embryonic stem cell pluripotency (33). Recent reports on differences in m6A modification in the roots, stems, and leaves of *Arabidopsis* have considered m6A as a key factor involved in the regulation of organ differentiation (17). Additionally, the genes consistently modified by m6A at all three ages have been reported to be mainly involved in regulating porcine liver cell differentiation and liver tissue development (30). Therefore, m6A might conservatively play a crucial regulatory role in the differentiation and development of animal and plant cells. Compared with the m6A peak abundance in each group and analysis using gene annotation, the GO analysis results revealed the RNA binding term in the three groups (Figures 3E–G); m6A exists widely in organisms and is involved in crucial biological functions. The main function of m6A is to regulate the fate of target genes by binding specific recognition proteins to target genes. In 2012, Dan Dominissini attempted to identify m6A recognition proteins in a MeRIP-Seq study by co-incubating m6A-modified RNA probes with mammalian cell lysates. The results indicated that the YTH family proteins YTHDF2, YTHDF3, and YTHDC1 specifically recognize and bind m6A sites in RNA. This protein family is also the largest known m6A recognition protein family (29). With an in-depth study on m6A, more recognition proteins will be discovered. Studies have shown that the competition between non-sense-mediated mRNA decay and sense-mediated mRNA decay is controlled by the preferential recruitment of ATP-dependent RNA helicases, such as UPF1 and UPF2, to target the mRNA. This activity is also involved in developmental processes such as myogenesis and adipogenesis (35, 36). Furthermore, possible interactions occur between m6a-mediated mRNA decay and other mRNA decay pathways. miRNA-mediated attenuation of mRNAs with long 3′ UTRs requires the combined action of Ago2 and the non-sense-mediated mRNA attenuation factors UPF1 and SMG7 (37). Therefore, we hypothesized that m6A-mediated mRNA decay plays a role in regulating the expression levels of key genes in yak skeletal muscle development.

Skeletal muscle development, a complex biological process, involves a multi-faceted network regulation. Research on the regulatory role of myogenic regulators and the regulation of skeletal muscle development by epigenetic modifications, including DNA methylation and histone modification, has expanded our preliminary understanding of the regulatory network of skeletal muscle development (38). Considering the role of m6A in regulating brain development and adipogenesis in mice, m6A was hypothesized to be involved in regulating skeletal muscle development (39–41). This study identified the pathways and genes involved in the functioning of negatively correlated m6A-modified genes. In the A vs. E group, the KEGG analysis explored the results of a conjoint analysis of MeRIP-Seq and RNA-Seq genes that participated in muscle development (e.g., AMPK and FOXO pathways) (42, 43). Fox family members, especially the *FOXO1* gene, are closely related to muscle growth and development as they play a pivotal role in myoblast fusion and muscle fiber-type transformation (42). In addition, AMPK not only plays a key regulatory role in muscle energy metabolism but also participates in the transformation of muscle fiber types (44). In our study, genes regulating AMPK and FOxO pathways were mainly expressed in adulthood, possibly indicating that muscle fibers in adulthood are regulated by these two pathways and thus proving that internal factors from a fetus to adulthood gradually transform a fast muscle into a slow muscle. According to the Venn diagram in Figure 5D, HES1 was negatively regulated in the three groups. In our experiment, the HES1 transcript in the skeletal muscle of the yak before birth (E group) exhibited a lower expression and a higher m6A chemical modification. HES1 protein, the most crucial effector molecule downstream of the Notch signaling pathway, is detected in almost all undifferentiated cells (45), mainly participating in the regulation of cell apoptosis, proliferation, and differentiation, especially of mammalian cells. Notch inhibits myoblast differentiation during the differentiation of muscle stem cells. One mode of action can be that activated Notch induces RBP-J to directly regulate Hes1 transcription, thereby inhibiting MyoD expression (46). Furthermore, NICD can be directly interact with the muscle differentiation factor, MEF2C, to regulate MyoD expression (47). These findings suggest that m6A modifications may perform an essential role in the development of yak LD.

## Conclusion

In conclusion, diverse m6A patterns were characterized in the genes expressed in the yak LD, which were found to be crucial regulators in the three developmental stages. A negative correlation between the m6A methylation level and the expression level of the modified genes revealed a major biological effect in negatively regulating adenosine methylation in transcriptional gene expression. The interaction between m6A-mediated mRNA attenuation and other mRNA attenuation pathways might be involved in yak skeletal muscle development. The m6A gene is mainly involved in regulating LD differentiation and development in yaks. This comprehensive map therefore provides a solid basis for determining the potential functional roles of m6A RNA modification in regulating yak muscle growth.

## Data Availability Statement

The datasets presented in this study can be found in online repositories. The names of the repository/repositories and accession number(s) can be found below: https://www.ncbi.nlm.nih.gov/, PRJNA751245.

## Ethics Statement

The animal study was reviewed and approved by the Animal Administration and Ethics Committee of Lanzhou Institute of Husbandry and Pharmaceutical Sciences of Chinese Academy of Agricultural Sciences (Permit No. 2019-002). All yaks were handled in strict accordance with good animal practices by following the Animal Ethics Procedures and Guidelines of the People's Republic of China. Written informed consent was obtained from the owners for the participation of their animals in this study.

## Author Contributions

PY and CL conceived and designed the experiments and explained the data. XM analyzed the main content of the data with the help of MC, XD, XW, XG, JP, and PB. YL performed the experiments with the help of PB. XM wrote the manuscript with the help of PY and CL. All authors contributed to the article and approved the submitted version.

## Funding

This study was funded by the Agricultural Science and Technology Innovation Program (25-LZIHPS-01), National Beef Cattle Industry Technology and System (CARS-37), and Herbivorous Livestock Industry and Technical System of Gansu province (GARS08). The Gansu Basic Research Innovation Group Program, Research on Innovation of Yak Molecular Breeding Technology (20JR5RA580), Science and Technology Aid Qinghai Cooperation Special Project (2020-QY-212), National Natural Science Foundation of China (32102524), and Natural Science Foundation Gansu province (21JR7RA032) also supported this study.

## Conflict of Interest

The authors declare that the research was conducted in the absence of any commercial or financial relationships that could be construed as a potential conflict of interest.

## Publisher's Note

All claims expressed in this article are solely those of the authors and do not necessarily represent those of their affiliated organizations, or those of the publisher, the editors and the reviewers. Any product that may be evaluated in this article, or claim that may be made by its manufacturer, is not guaranteed or endorsed by the publisher.

## Supplementary Material

Supplementary Figure 1 A correlation between qRT-PCR and RNA-Seq data. The trend lines and formula in each scatter plot represent correlation coefficients.

Supplementary Table 1 Primers for quantitative real-time polymerase chain reaction (qRT-PCR).

Supplementary Table 2 Summary of sequenced and mapped data of the methylated RNA immunoprecipitation sequencing (MeRIP-Seq) and input RNA sequencing (RNA-Seq) samples.

Supplementary Table 3 Summary of MeRIP-Seq data and reads mapped to the yak reference genome.

Supplementary Table 4 Peak detection number statistics.

Supplementary Table 5 Kyoto encyclopedia of genes and genomes (KEGG) enrichment of consistently modified genes in yak longissimus dorsi (LD) at the three developmental stages.

Supplementary Table 6 Differentially methylated N6-methyladenine (m6A) genes enriched in the gene ontology (GO) and KEGG analyses of each group.

Supplementary Table 7 GO enrichment and KEGG pathway enrichment analyses of each group.
