# Supplementary figures and images for: Regulatory Role of N6-Methyladenosine in Longissimus Dorsi Development in Yak
Source: Front Vet Sci. 2022 Apr 13;9:757115. doi: 10.3389/fvets.2022.757115 (PMC9043854; doi:10.3389/fvets.2022.757115)

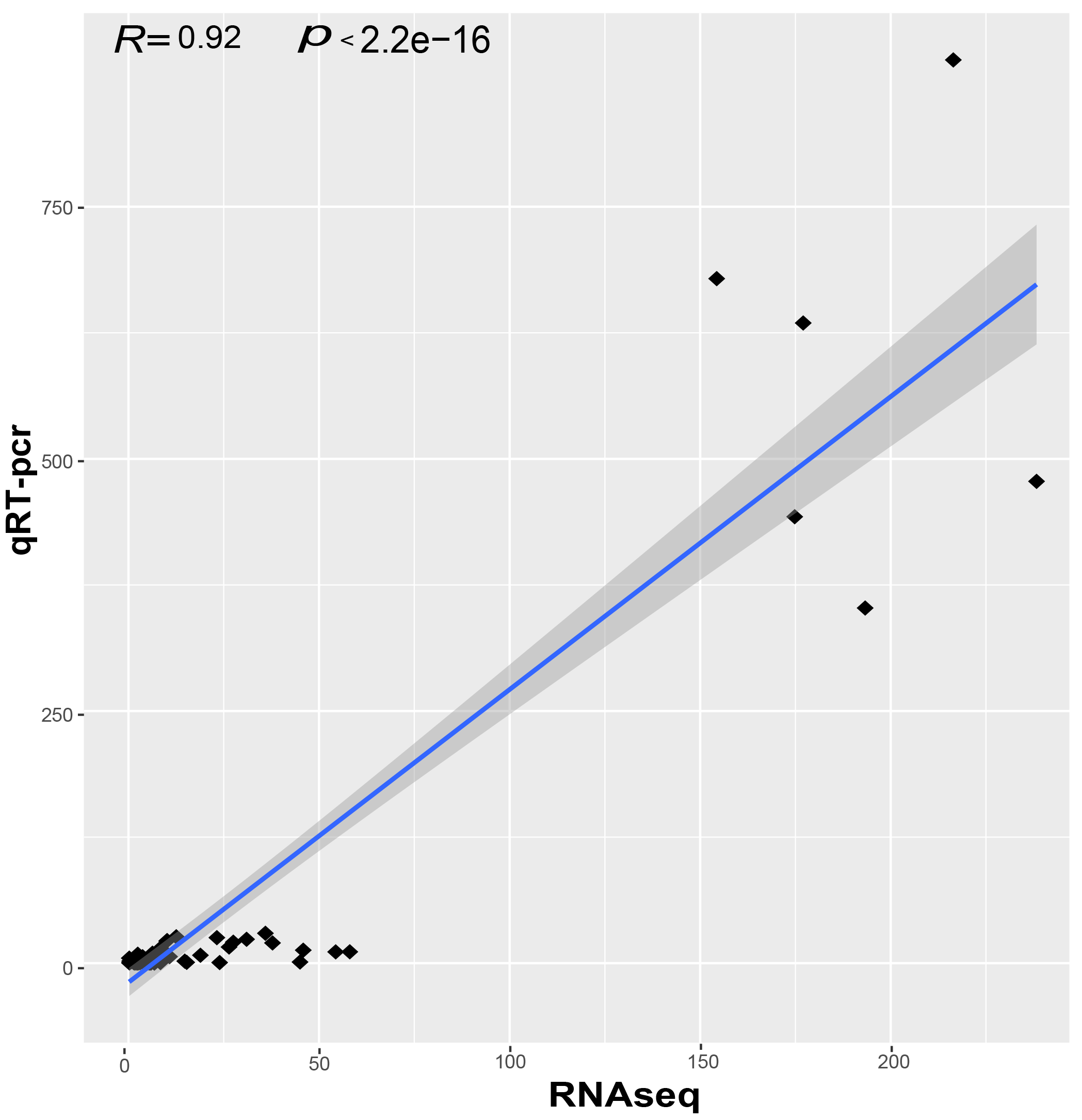

Supplement: Supplementary Figure 1 — A correlation between qRT-PCR and RNA-Seq data. The trend lines and formula in each scatter plot represent correlation coefficients. [file Image_1.TIF]
